# Employing a Participatory Research Approach to Explore Physical Activity among Older African American Women

**DOI:** 10.1155/2014/941019

**Published:** 2014-08-21

**Authors:** Emerson Sebastião, Kelechi Ibe-Lamberts, Julie Bobitt, Andiara Schwingel, Wojtek Chodzko-Zajko

**Affiliations:** Aging and Diversity Lab, Department of Kinesiology and Community Health, University of Illinois, 209 Louise Freer Hall, 906 S Goodwin Avenue, Urbana, IL 61801, USA

## Abstract

*Introduction*. Older African American women are particularly vulnerable to unhealthy lifestyle behaviors such as physical inactivity and the resultant chronic diseases and conditions. This study explored older African American women's perception of physical activity as well as facilitators of and barriers to being physically active in their local environment. *Methods*. Using a participatory research approach, a total of 7 women aged 65 years and over had their PA level assessed objectively through accelerometry. In addition, physical activity was discussed through the photo-elicitation procedure, which was supplemented by semistructured interviews. Qualitative thematic analysis was used to identify patterns and themes emerging from participants' interview. *Results*. Participants exhibited low levels of physical activity and viewed “physical activity” to be a broadly defined, nonspecific construct. Interviews revealed that many participants lack important knowledge about physical activity. A variety of personal, social, and environmental facilitators and barriers were reported by the participants. *Conclusion*. Efforts should be made towards clarifying information on physical activity in this population in order to help them incorporate physical activity into their routines, overcome barriers, and make use of opportunities to be active.

## 1. Introduction

The exponential growth in numbers of older adults is unprecedented in the history of the United States. Longer life spans and aging baby boomers will double the population of Americans aged 65 years or older during the next couple of decades to about 72 million. Thus, it is expected that, by 2030, older adults will account for nearly 20% of the United States population. Currently, there are approximately 40 million persons aged 65 years and over in the United States. This represents 13% of the United States population, meaning that 1 in every 8 Americans is in this age group [1].

Along with the general aging trends for America's population, a significant increase in racial and ethnic diversity will be also observed. Although young individuals in the United States current reflect diversity more strikingly than their older counterparts, the racial and ethnic makeup of older adults is also changing [1]. African American population is living longer with African American women living longer than men. The African American older population was about 3.3 million in 2010 and is projected to grow over 9.9 million by 2050. Currently, African Americans aged 65 years and over account for approximately 8% of the older population; however, this number is expected to increase to 11% by the year of 2050 [2, 3]. Population aging comes with wide-ranging challenges, not only for the individual, but also for society, especially for the health sector. Expenditures for health care are expected to increase at considerable levels as chronic diseases increasingly affect the growing numbers of older adults in most developed countries [1, 4]. High rates of chronic diseases and disabilities are observed in the older population as recently demonstrated by a Centers for Disease Control and Prevention report [1]. African Americans bear a disproportionate burden of disease morbidity, disabilities, and injuries compared to other racial groups [5]. Health disparities among underserved groups are well documented in the United States. For instance, information shows that in the United States nearly 30% of the population aged 65 years and over has diabetes. Among them, African Americans (18.7%) present the highest prevalence compared to Whites (10.2%) [6]. In addition, other health issues also appear to be disproportionately prevalent in the African American population. For example, African Americans exhibit higher level of disabilities compared to Whites [7, 8]. Different factors, including physical inactivity, have been suggested to explain health disparities, such as higher rates of diseases in African Americans [9–12]. African American women are a particular vulnerable group to unhealthy lifestyles, such as physical inactivity, and the resultant chronic diseases and conditions.

Physical activity has the potential to promote health and well-being, delay the onset of chronic diseases and disabilities, and reduce the risk of premature death [13, 14]. However, for health benefits, it is recommended that adults accumulate at least 150 minutes per week of moderate to vigorous intensity physical activity [15]. Evidence-based interventions focused on lifestyle behavior changing, such as physical activity promotion, have been recommended to enhance health in different age groups including the older adult population [16]. Despite solid evidence of the benefits of physical activity, participation rates are low worldwide with the older population reporting higher levels of inactivity compared to young age groups [17]. However, data from the Behavioral Risk Factor Surveillance System shows that in the United States African American present with very low rates of physical activity participation at recommended levels [18]. Additionally, older African American women appear to be a vulnerable group to physical inactivity [19]. Factors such as health issues, family demands, lack of money, reduced public resources available, and neighborhood safety have been suggested as factors preventing individuals from engaging in physical activity among African Americans and other at risk population groups [20, 21].

However, it is also possible that the lack of physical activity among older African Americans could be related to public health messages. Public health messages used to educate the general public towards physical activity may not be clearly interpreted by, or are not adequately reaching this population [22]. As interventions should be culturally tailored, so should the messages used to promote positive behaviors such as physical activity. Thus, it is essential to first understand how a certain group of interest views a given topic. In this sense, a study aiming to explore how older African American women conceptualize physical activity is of great importance and is a step towards the development of culturally sensitive messages that may have a better impact on this population. Furthermore, cultural values and the legacy of racism, segregation, and discrimination carried by this population may shape their views of opportunities for and barriers to physical activity.

By employing objective assessment of physical activity in conjunction with a participatory research approach, this study explored the understanding of physical activity among inactive older African American women. Moreover, facilitators of and barriers to being physically active in their local environment were also examined. Physical activity is a complex and dynamic behavior that clearly depends on multiple factors as previously mentioned. In this sense, theoretical frameworks are important tools to help investigators better understand and interpret their findings. Ecological models are adequate and effective for better understanding of complex health behaviors that are involved in daily living, such as physical activity. This study was driven by the Social Ecological Model of Health Behavior [23] which suggests that individual behavior does not occur in isolation but emerges from a complex multi-level interaction between the individual, the environment, and the social cultural context where they live. This model provides a useful and important framework for understanding the numerous factors enabling or detracting from participation in this complex and dynamic behavior.

## 2. Methods

### 2.1. Study Design

A cross-sectional mixed-methods research design approach employing a participatory research technique was adopted. The Institutional Review Board of the University of Illinois approved the research protocol and signed inform consent was obtained from participants before data collection.

### 2.2. Sample and Setting

A total of 7 older African American women aged 65 to 75 years living in Urbana-Champaign, IL, were interviewed. The recruitment process involved flyers posted in health centers, senior centers, and local food stores. This procedure was conducted alongside the engagement of community leaders. To be included, participants had to be female and self-identify as African American. Additionally, participants were aged 60 years and older and lived in the community. Participants had to be able to walk independently and to be able to answer questions coherently by themselves. This study did not include older adults living in institutionalized settings (i.e., nursing homes, etc.), as well as individuals with significant cognitive and physical functioning decline. Participants selected to take part in this study were cash compensated throughout each step of the study.

### 2.3. Data Collection

Participants in this study followed three different steps during the data collection process that occurred during October and November of 2013.

#### 2.3.1. Sociodemographic and Health Data

Participants completed a questionnaire to collect information on age, height, weight, years of education, income level, marital status, self-reported health, number of diagnosed diseases, medications taken, and health insurance coverage. At this phase, participants could select whether to meet at the investigators laboratory/office or at their own home.

#### 2.3.2. Accelerometry and Camera

Participants wore an accelerometer at the hip (Actigraph GT3x plus) for 7 consecutive days for physical activity assessment. Participants were asked to wear the device for at least 10 hours each day. Additionally, participants were instructed to maintain their normal routine.

Along with the week wearing the accelerometer, participants were given a disposable camera to take pictures of meaningful places (indoor and outdoor) that they go and things they do in their daily lives, as well as people they interact with, which for them were related of being active or physical activity. Participants were instructed to represent both week and weekend days in their pictures. This participatory research approach is known as photo* elicitation* [24]. Photo elicitation is a participatory research technique that invites participants to take photographs of salient features in their lives that are personally meaningful and possess significant explanatory power. After completion, accelerometers and cameras were retrieved for physical activity data analysis and photo development. Participants in this study received previous training on how to handle the devices.

#### 2.3.3. Photo Elicitation Plus Semistructured Interview

After photo development, participants were invited to take part in the individual interview, which was conducted in a quiet room (investigator's office). Among approximately 25 photos, 6 were selected to be part of the interview process. Four meaningful photos were selected by the participants and 2 additional ones were selected by the investigator. The 2 photos selected by the investigator were selected with the goals of providing different insights from the ones selected by the participant, thereby assisting with the physical activity discussion. Interviews were conducted in English and lasted about 60 minutes. During the interview, participants were asked questions about the photos and their representation. Additionally, follow-up questions were used to discuss physical activity (interview guide available upon request with the investigator). At the end of each interview the entire set of photos provided by the participant were displayed in the table. Follow, participants were asked to take one more look on the entire set of photos to talk about anything that was not previously covered in the interview, if any. The interviews were recorded using a digital recorder and transcribed to a word document by native English speakers.

### 2.4. Data Analysis

The data collected from the questionnaires were analyzed using descriptive data. Accelerometer data were analyzed using ActiLife 6.0 software employing the Freedson et al. adult cut-score equations [25]. The 150 minutes per week of moderate to vigorous physical activity intensity was adopted as the cut point to classify the participants as active or inactive for health benefits [15].

Qualitative data were analyzed using a descriptive thematic approach. The 6 phases described by Braun and Clarke [26] were adopted during the analysis. Thematic analysis consists of the identification of themes that naturally emerge from the data as being important to the description of the phenomenon. Therefore, after transcription, the interviews were coded and analyzed for common themes and key patterns that emerged from the conversations. Credibility and reliability of the data were examined employing investigators' triangulation with 2 other researcher colleagues. A hundred percent agreement was found between the investigators involved in this process.

## 3. Results

**Figure 1:**
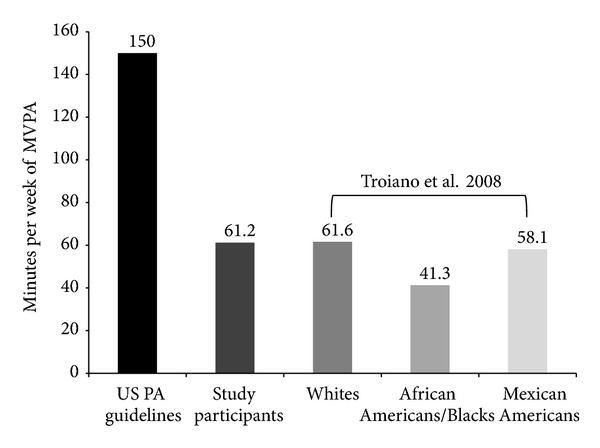
Level of moderate to vigorous physical activity (MVPA) recommended by the current United States physical activity guidelines, mean level of MVPA observed in the older African American women participating in this study (*n* = 7) living in Urbana-Campaign, IL, and mean level of MVPA observed in the national sample of older women (60 and plus) reported in the Troiano et al. study [19].

Seven older African American women took part in this study (68.43 ± 3.20). All participants were classified as inactive for health benefits according to the criteria adopted above (Figure 1).  Table 1 displays, in detail, the sociodemographic and health information of the participants.

Regarding habitual physical activity, accelerometer data showed that none of the 7 participants achieved the recommended level of physical activity for health benefits. Figure 1 displays the current recommendations for physical activity to achieve health benefits, the mean level of physical activity observed in the participants of this study, and the findings observed by Troiano and colleagues [19] study conducted in a national sample in the United States.

After completion of the interviews, the following themes emerged.

### 3.1. “Physical Activity” Defined as Broad Concept

The women in this study viewed physical activity as a broad concept, which extends beyond traditional exercise routines. Although they did not provide a clear definition of what physical activity is, some of the participants were able to suggest examples of what physical activity means to them. The way the older inactive African American women conceptualized physical activity was generally consistent with a public health definition of physical activity, that is, covering a variety of lifestyle-related activities. The following is an example:

*“…it is like getting out there and mowing the grass; getting out there and cleaning the gutters, painting like I paint. And that is what I think, and apart from that is exercising too.” (Female 71)*

### 3.2. How Much? As Much as You Can

When asked about how much physical activity women in their age should do, and if they ever heard about any government recommendation regarding physical activity, the answers were diverse. The United States guidelines recommend that every adult should accumulate at least 150 minutes per week of moderate to vigorous physical activity for health benefits. However, none of the interviewed women appeared to be aware of the current recommendations. Although they all agreed that being physically active was important, there was a misconception regarding the amount needed. In addition, none of the women talked about intensity, which is an important element within the recommendation along with frequency and duration to achieve health benefits. The following is an example:

*“…As much as they can… you do what you can! But be consistent you know?… Even if you have a walker, you do that. This is just my opinion.” (The same woman said) “… Twenty, ten minutes every morning of physical exercise and that is all the government requires. But that is like selling a product or something so I do not know the validity of those type [sic] of things.” (Female 71)*

The interviews revealed that despite agreeing that some physical activity is better than nothing, women in this study understood little about the specific details of physical activity recommendations in terms of frequency, duration, and intensity.

### 3.3. Facilitators of and Barriers to Physical Activity

#### 3.3.1. Social Facilitators

The older African American women described different factors related to social support, that is, friends; senior centers and churches as facilitators for physical activity. Women talked how having a companion for physical activity could be an encouragement to be active. The following is an example: “*…a buddy system is one of the most effective ways of getting people involved… It takes a different type of courage and strength for people to go and do something…” (Female 65).* Community-based institutions were also viewed as facilitators of physical activity. Women described how they use the community center as a place to be active. For example:* “…most of the time I try to go down to Douglas Center at least for 45 minutes a day maybe not often [sic] Fridays but most of the time for about 45 minutes… The senior building (Douglas Center Annex) in the mornings I would walk over there for, [sic] maybe a half-hour to 45 minutes…” (Female 66)*. Moreover, the interviews revealed that church initiatives towards physical activity may be a promising way to promote physical activity among African American women. The following is an example:* “From time to time, we have people. We have students that come through…we have people who come through and we try to do some programming…one young man that comes through and he was teaching us tai chi. He taught tai chi to us and it was kind of fun. So we do have things there…” (Female 68)*.

#### 3.3.2. Environmental Facilitators

Participants recognized the importance of green areas and parks nearby as facilitators for physical activity. The following is an example:* “…and this is like in the back of my house… you know there is a big field and there is like a walkway through the park district… they put walkways and bikeways. I just like the nature, it just lifts you up, it is just beauty. It is a beauty of nature and it makes you…eyes get in tune with nature. I love to hear the birds, I love to hear the crickets, I love all of that; the creatures that are on the earth, now does that make sense?*”* (Female 67).*

#### 3.3.3. Personal Barriers

African American women participating in this study talked about how health problems related to having a chronic physical condition prevent them from being more active. One woman describes her experience when inviting a friend to walk together:* “…Oh need a new knee. I need a new knee replacement *(female talking about reason on* [sic] *why older African Americans are not so active).* I am trying to get my friends to walk more, like I tell my friend [She] now she had a knee replacement and she will say (“You know I cannot walk”)” (Female 66).*

#### 3.3.4. Environmental Barriers

Environmental barriers emerged in terms of bad weather and lack of safety in the environment, that is, cracked sidewalks, and neighborhood in the context of fear of crime. Women expressed how the cold weather hinders them from being more active. The following is an example:* “…So it is a lot of walking area; and then sometime I come back down Kirby. So I just… Like I said I like walking; but I do not like walking in the cold…” (Female 66)*.

In addition, problems in the neighborhood related to lack of infrastructure such as poor sidewalks and fear-related violence were expressed as factors detracting participants from being more active. The following is an example:* “You see this here (… talking about a cracked sidewalk). I almost tripped and fell on my face. My friend … actually did that and she had to go to the doctor and they had to do a MRI. She fell flat on her head. That is like about 2 inches up and if you are walking and you are not looking, anybody not even an old person but even a child could fall and hurt themselves” (female 67)*; and* “When it was nice weather, they have their hoods on their heads. So I got that feeling that something was not quite right if you cover it up, you know. I just feel like it was not really safe anymore. Cause I used to walk around [sic].” (Female 68)*.

Table 2 summarizes the facilitators and barriers influencing physical activity that emerged from participants' photos and interviews.

## 4. Discussion

Factors influencing physical activity in different age groups have received increased attention due to concerns regarding physical inactivity and its negative impact on individual health and cost to the society. Few studies have examined perceptions of physical activity among older African American women. This study adopted a mixed-methods approach employing a participatory research technique to explore perceptions of and factors influencing physical activity among older African American women.

It was observed that, on average, women participating in this study perform nearly 61 minutes per week of moderate to vigorous physical activity. This value is less than half of the current recommended level of physical activity to achieve health benefits [15]. However, this value is higher than those reported by Troiano and colleagues [19]. These authors observed in a national sample of older adults in the United States that African Americans/Blacks aged 60 years and over perform on average nearly 41 minutes per week of the recommended level of physical activity. However, the values observed for the women in this study are similar to the values observed by Troiano and colleagues when examining physical activity in older White and Mexican American women of similar age. The averages observed were, respectively, 61.6 and 58.1 minutes per week. Taken together, these findings suggest that physical inactivity continues to be a concern in the older adult population in the United States. In this sense, urged public health actions are needed to mitigate such problem.

Furthermore, it was observed that many participants lack basic knowledge regarding recommended levels of physical activity, especially in terms of frequency, duration, and intensity. Moreover, it was observed that women in this study believe that the amount of physical activity needed for people in their age should be determined by the individual. These findings are similar to those observed by Wilcox et al. [27] in a study of underactive rural women living in South Carolina. Similar to our findings, the authors also observed that their participants held the opinion that frequency, duration, and intensity should be determined by the individual. Taken together, these findings suggest that the promotion of the Physical Activity Guidelines for Americans should be stressed in order to make individuals more aware of the scientific evidence regarding the quantity and intensity of physical activity needed to achieve health benefits. Even though attempts to disseminate information about the Physical Activity Guidelines for Americans [15] exist (e.g., websites), none of the participants in this study heard about any government recommendations for physical activity. Although all women in this study agreed that some physical activity is better than none, the findings suggest that none of them appear to be fully aware of some of the basic recommendations for physical activity to achieve health benefits.

Facilitators of and barriers to physical activity were also explored. Our in-depth interviews suggest that social and environmental factors often serve as facilitators of physical activity in this population. Regarding barriers, a number of personal and environmental factors were found to be barriers to physical activity. These findings are similar to those observed by Mathews et al. [20] in a multicultural study conducted among older adults as well as several other studies in the literature [20, 28–31]. For instance, Mathews and colleagues [20] examined physical activity enablers and barriers in a sample of older Americans composed of 6 racial/ethnic groups (i.e., African Americans, American Indians, Latinos, Chinese, Vietnamese, and Whites). The authors observed that no barriers to physical activity were mentioned in common across all groups; however, barriers discussed frequently by at least 3 racial/ethnic groups included physical health problems, built environment/community design, financial costs, weather, and lack of time. Common facilitators across 5 or more groups were observed by the authors who found that social support, conducive built environment, health benefits, and physical activity programs were reported as physical activity enablers by the participants. Belza and colleagues [21] also found similar findings for both enablers of and barriers to physical activity studying the most prevalent racial/ethnic groups of older adults living in United States. Taken together, the findings from these studies allied to the findings of the present study suggest that most of the barriers and facilitators for physical activity are not exclusive for African Americans. Similar factors positively and negatively affecting physical activity participation have been reported by other racial groups. These findings reinforce current knowledge of factors associated with physical inactivity in older African Americans and older adults in general and constitute important implications for public health initiatives and community-based interventions aiming to promote active living among African Americans.

Both facilitators and barriers related to physical activity expressed by the women participating in this study are in line with proximal and distal factors influencing physical activity proposed by the Social Ecological Model of Health Behavior. Based on the information gathered from the participants, it is noteworthy to highlight the role of the organizational level in both promoting and preventing physical activity. It reinforces the necessity of developing community-based interventions to physical activity to target specific groups.

The present study employed a participatory approach known as photo elicitation along with an objective assessment of physical activity to enhance our knowledge of how physical activity is understood among older inactive African American women. The combination of objective (accelerometry) and subjective (photo elicitation) approaches provides an opportunity to add to the extant literature. Previous studies addressing similar purposes as the present study adopted only focus group or individual interviews along with self-reported or subjective assessments of participant physical activity levels. Since individuals often overestimate their physical activity levels when assessed by self-report methods [32], it is difficult to be confident about their actual activity status. The combination of both qualitative and quantitative approaches has the potential to generate more meaningful and useful information. It is important to highlight that photo elicitation is not a substitute for other methods such as individual interviews or focus group. Instead, the technique serves as a useful supplement to these approaches that have the potential to enhance the quality of the findings. As stated by Hagedorn [33],* “…the images captured in photography invite people to take the lead in inquiry, facilitating their discussion of an experience. Photographic interviews elicit a unique return of insights that might otherwise be impossible to obtain with other techniques. Photographs sharpen memory and give the interview an immediate character of realistic construction and function.”* Moreover, investigators state that the act of sharing and talking about pictures evokes the power of visual images to communicate not only life experience but individuals' expertise and knowledge. Thus, although photo elicitation has the purpose of having people recording and reflecting positive and negative things in their community, it also promotes a critical dialogue about important community issues by the lens of large and small group discussion of photographs; and also can be used as a tool to reach policymakers [34, 35].

Our findings must be interpreted with care due to some limitations. Our study was based on participatory-approach along with in-depth interview of a nonrandomized sample of 7 inactive older African American women. We do not know if the patterns that emerged would have differed if a different cohort of African American women had been selected. Caution is warranted when generalizing the outcomes to the broader population of older African American women and African Americans in general. However, despite the small sample size, it is important to highlight that qualitative studies drastically differ from quantitative studies regarding “ideal” sample size. Moreover, studies adopting partial or full qualitative designs are less concerned with the generalizability of the findings. Instead, the goal is to better understand how particular groups view/perceive a particular phenomenon and extract rich data that can be add to the literature.

Although no amount of physical activity can stop the aging process, there is now compelling evidence that a moderate amount of regular physical activity can minimize the physiological effects of aging and increase active life expectancy by limiting the development and progression of chronic disease and disabling conditions [36]. Culturally, sensitive approaches to physical activity promotion are essential to help reduce heath disparity among older adults. Thomas et al. [37] suggest that it is important to match the cultural characteristics of minority populations with public health interventions to enhance receptivity to, acceptance of, and salience of health information and programs. The authors recognize the importance of factors like belief systems, religious and cultural values, life experiences, and group identity as powerful filters through which information is received. Therefore, such factors should be considered in the development of both interventions aiming to improve chronic diseases through physical activity as well as health communication campaigns. In fact, health communication is believed to be an important aspect for educating and promoting positive behaviors such as physical activity at the population level. As noted by Balbale et al. [22] tailoring and improving the message design for underserved groups is essential if we are to mitigate or eliminate the burden of health disparities.

## Figures and Tables

**Table 1:** Sociodemographic and health information of older African American women (*n* = 7) living in Urbana-Campaign, IL.

Variables	Participants (*n* = 7)
Age, mean (SD)	68.43 (3.20)
Education	
High school or less	1
Incomplete college	4
College	2
Marital Status	
Divorced/separated	5
Widowed	2
Annual income	
$10,000–$14,999	2
$15,000–$19,999	1
$20,000–$29,999	3
$50,000 and over	1
Chronic diseases	
0-1	5
2 or more	2
Medication	
0-1	5
2 or more	2
BMI, kg/m^2^, and mean (SD)	28.64 (5.74)
Self-reported health, (very good)	7
Health insurance, (yes)	7
Do you think that a person at your age can improve your health through regular exercise or balanced diet or by stopping smoking? (yes)	7

**Table 2:** Summary of the facilitators of and barriers to physical activity that emerged from the interviews with older African American women (*n* = 7) living in Urbana-Campaign, IL.

Category	Factors influencing physical activity
Facilitators	(i) Friends (ii) Senior center (iii) Church; (iv) Green area and parks
Barriers	(i) Health issues (ii) Bad weather (iii) Unsafe environment
